# Homœpathic Brigade Surgeon

**Published:** 1861-10

**Authors:** 


### HOMŒPATHIC BRIGADE SURGEON.

We notice the following item in the Daily Courier, and similar ones in some of the other daily papers:

"Appointment.—We are pleased to learn that Dr. H. C. Blanchard has been appointed by General Scroggs, Surgeon of the Eagle Brigade, and will be at the head of the medical department of that organization. The appointment, to use a hackneyed phrase, is one eminently "fit to be made.""

Perhaps we do not fully understand this matter, since this is said to be a *Independent Brigade;* but we suppose such appointment cannot be made until after an examination by the United States Army Medical Board and that appointment is from the United States Government through the Secretary of War. Brigade Surgeons are not appointed by the State Governors, as is the case with the Regimental Surgeons. However this may be, we hazard the opinion that in this case an examination will never be requested. The object will be fully accomplished when the *appointment* and *resignation,* on account of *extensive private practice* shall have been duly noticed by the daily press. It is designed only for the climate of Buffalo—*for home influence.*

There have been but two or three Homœpathists, thus far, admitted to practice, in the Volunteer Army, and these, we are told, are the most unhappy men in the United States—were admitted to examination by the Board, under false pretense of being regular physicians, and are the last men who can expect any consideration from the Army Medical Staff.

Of this rumored appointment to the Eagle Brigade, we can only say, We have not *seen* the Brigade, or heard of any man who has. We have not known of any physicians and surgeons, who are capable of doing the duty, who would be likely to serve as Regimental Surgeons, and Assistant Surgeons, provided the appointment of any Homœpathist as the Brigade Surgeon is confirmed.

If such Brigade should ever be seen, and should be officered in homœpathic style, and the soldiers should at any time require important surgical operation or attendance, we can only pray, God have mercy on their souls.

Touching the point of capacity, we are encouraged to hope some thing in the future never realized in the past, since the candidate for distinction assures his neighbors that, in future, "he is going to pay a devilish deal more attention to surgery, and a devlish deal less to the *small pills.*"

Now, since the renunciation of Homœpathy is becoming common and fashionable, we fear there may be a *violent stampede,* and desire, in behalf of the profession, to protest against their coming "our way." If Homœpathy can no longer claim their attention, and, with Dr. Peters, they "*painfully and reluctantly endeavor to cast their lot with other friends, other theories, and other practice, feeling absolutely degraded when making, what they consider, necessary trials,*" perhaps laying on hands, repeating charms, or operating in free love sympathies, may offer inducements which will be acceptable. Honest, scientific, common sense practice of medicine, is altogether unsuited to their habits and capacities.

